# Targeting T Cell Immunometabolism for Cancer Immunotherapy; Understanding the Impact of the Tumor Microenvironment

**DOI:** 10.3389/fonc.2014.00107

**Published:** 2014-05-16

**Authors:** Mary B. Mockler, Melissa J. Conroy, Joanne Lysaght

**Affiliations:** ^1^Department of Surgery, Trinity Centre for Health Sciences, St. James’s Hospital, Trinity College Dublin, Dublin, Ireland

**Keywords:** immunometabolism, cancer, T cells, metabolic targeting agents, obesity

## Abstract

The immune system has a key role to play in controlling cancer initiation and progression. T cell activation, which is central to anti-tumor immune responses, coincides with changes in cellular metabolism. Naïve T cells predominantly require an ATP generating metabolic profile, whereas proliferating effector T cells require anabolic metabolic profiles that promote rapid growth and proliferation. Furthermore, specific T cell subsets require distinct energetic and biosynthetic pathways to match their functional requirements. The often hostile tumor microenvironment can affect T cell immune responses by altering the resulting cellular metabolism. Tailoring immune responses by manipulating cellular metabolic pathways may provide an exciting new option for cancer immunotherapy. T cell responses might also be skewed via metabolic manipulation to treat the complications of obesity-associated inflammation, which is a rapidly growing global health problem and a major risk factor for many malignancies. In this review, the diverse metabolic requirements of T cells in anti-tumor immunity are discussed, as well as the profound influence of the tumor microenvironment and the possible avenues for manipulation to enhance anti-tumor immunity.

## Introduction

A unique bioenergetic challenge is faced by cells of the immune system when they recognize mutated or over expressed tumor antigens presented by antigen presenting cells. They must rapidly proliferate with a specific phenotype and function to eradicate tumor cells (1). The ability of a T cell to transition from a naïve, to effector, to a memory phenotype is determined by metabolism and the metabolic program varies to match the T cell subset in order to enable cell survival and function (2). Activated T cells switch from naïve T cells, mainly requiring generation of ATP and replacement biosynthesis (3) to effector T cells (T_EFF_), which must rapidly increase cell mass and elicit effector functions through cytokine production and cytotoxicity (4). This switch from primarily oxidative phosphorylation (OxPhos) to glycolysis supports the demands of rapid cell growth. Primary fuels and amino acids, such as glucose and glutamine must be available to support these distinct metabolic pathways (5, 6). Unavailability of these fuels occurs during malnutrition and can lead to immunosuppression (7), while excess fuel in diet-induced obesity is associated with a constant low level state of inflammation (8). We have previously identified T cells as key players in adipose tissue inflammation in obesity-associated cancer and here, we suggest that their metabolism might be targeted to control chronic inflammation and lessen the clinical manifestations of obesity (9). In comparison, the long lifespan of memory T cells (T_MEM_) poses a different metabolic demand and to facilitate this need, T_MEM_ cells utilize fatty acid oxidation (FAO) as their main energy source (10). The balance between different T cell subsets can be altered by multiple environmental factors in the tumor resulting in the activation of metabolic pathways and the establishment of inappropriate T cell responses. Direct manipulation of T cell metabolism has the potential to provide a new avenue for cancer immunotherapy and obesity-associated inflammation and this will be discussed.

## How T Cell Metabolism Affects Function

**Figure 1:**
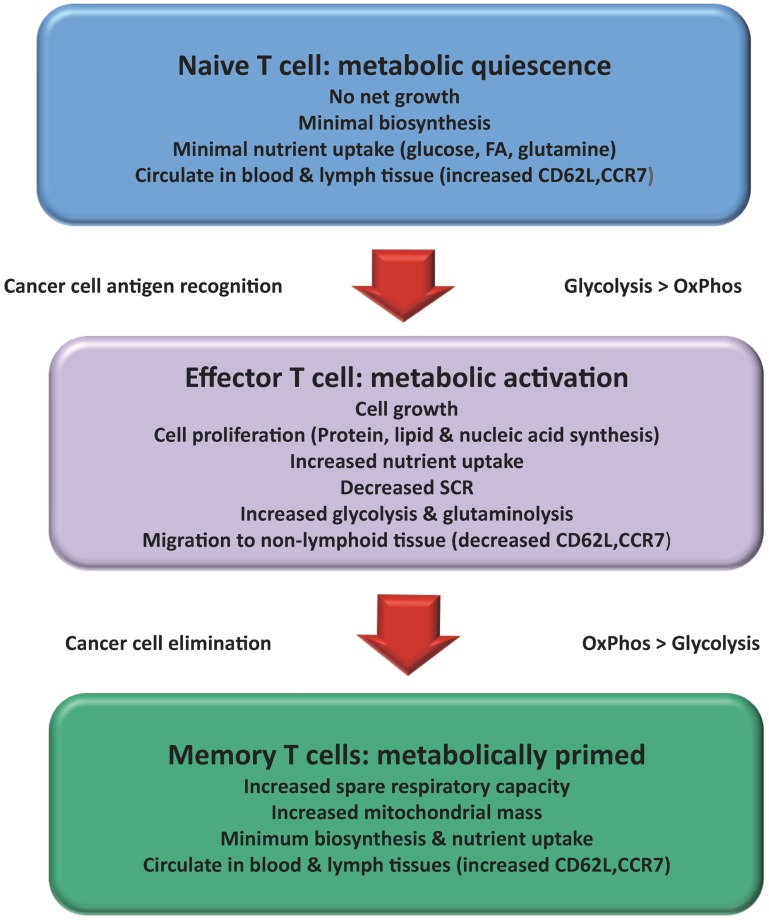
**Changes in T cell metabolism are related to function: Over the course of an immune response the metabolism of a T cell changes**. Naïve T cells mainly used a mixed fuel OxPhos to produce ATP. Once activated the T cell adopts a metabolic profile that is similar to many cancer cells (Warburg effect). Consumption of glucose and glutamine increases to support increased cell growth and proliferation. A subset of activated T cells survives to become T_MEM_ reverting back to lipid oxidation with increased capacity for efficient energy generation and survival. Activation of T cells results in changes in migration patterns to ensure they can successfully migrate to areas of inflammation and cancer.

At each stage of a T cell’s development, its metabolism must support the function of the cell through the provision of energy and biosynthetic precursors (11) (Figure 1). It is vital that T cells undergo the appropriate activation and differentiation to ensure that homeostasis is maintained (12).

### Naïve T Cells

The primary function of naïve T cells is antigenic surveillance, which requires relatively small amounts of ATP to support processes such as ion homeostasis, membrane integrity, and rearrangement of the actin cytoskeleton for movement (13). Thus naïve T cells have a catabolic metabolic profile that favors energy production over biosynthesis (14). It is estimated that 96% of ATP in naïve T cells is generated through OxPhos, with the remaining 4% being generated by glycolysis (3). It is an oxygen dependent process and produces 36 molecules of ATP per glucose molecule (1). Naïve T cells require extrinsic signals to maintain this basal metabolism. Interleukin-7 (IL-7) regulates glucose uptake via the cell surface trafficking of Glut1, which is modulated by the PI3K/Akt/mTOR pathway (15). This regulation of glucose uptake by IL-7 is critical to maintain homeostasis and to prevent apoptosis by the pro-apoptotic protein Bim (16).

### Activated T Cells

In contrast, T_EFF_ cells adopt an anabolic metabolic profile to meet the increased energy requirements associated with cell growth, proliferation, differentiation, and effector functions (4). T_EFF_ cells utilize aerobic glycolysis even though it is less efficient than OxPhos yielding only four moles of ATP per glucose molecule (17). Aerobic glycolysis provides advantages over OxPhos in rapidly dividing cells; firstly, metabolic intermediates needed for cell growth and proliferation are generated by aerobic glycolysis (11). Secondly, increased OxPhos results in increased production of reactive oxygen species (ROS), which can lead to apoptosis of T cells (18). Thirdly, aerobic glycolysis produces ATP faster than OxPhos. Therefore, once glucose is not a limiting factor, the pace of glycolytic flux will produce enough ATP to meet the energy demand of the T_EFF_ cells (19). Finally glycolysis is not oxygen dependent and can accommodate T cell survival in a hypoxic environment, a common feature of many solid malignancies (20). OxPhos is still utilized in T_EFF_ cells however aerobic glycolysis is the main pathway for glucose metabolism (21). Activated T cells must engage distinct transcriptional programs to both establish their differentiation status and elicit the effector functions required (22). These changes are similar to those observed in rapidly dividing tumor cells with many of the same metabolic regulators involved, including mTOR (23). The main transcription factors up-regulated by mTOR are c-Myc and hypoxia-inducible factor-1α (HIF-1α) and it appears that only c-Myc is necessary for the glycolytic switch that occurs during early T cell growth and proliferation. mTOR dependent up-regulation of c-Myc in the first 24 h following T cell activation is crucial in the transition from a naïve T cell to a T_EFF_ cell (21). Effector CD4^+^ T cells can be distinguished from effector CD8^+^ T cells due to their ability to increase OxPhos by up to two-fold after activation, whereas activated CD8^+^ T cells do not increase OxPhos above that of resting cells (24). This results in activated CD4^+^ T cells having a survival advantage over CD8^+^ T cells when pro-glycolytic signals are limited (25). CD8^+^ T cells execute the majority of immune-mediated tumor killing through the release of cytolytic factors, such as perforin and granzymes (26). Depending on the polarizing signals in the tumor microenvironment the different CD4^+^ T cell subsets can promote or suppress a defensive host response to cancer (27). Each CD4^+^ T cell subset has a unique metabolic profile, which is adapted to its function with Th1, Th2, and Th17 cells displaying a glycolytic metabolic phenotype (28). In contrast to Th1 and Th17 cells, Th2 cells readily develop in the absence of mTORC1 but fail to develop in the absence of mTORC2 (29). T_REG_ cells unlike other T_EFF_ cells mainly use lipid metabolism and have elevated levels of AMPK activation (28). The utilization of lipid oxidation by T_REG_ cells might play a central role in their survival advantage over T_EFF_ cells and in the maintenance of a stable pool of pro-tumor immune cells (30).

One area that currently warrants further investigation is how metabolism affects, and is affected by migration of activated T cells. Migration from nutrient and oxygen rich regions of the peripheral blood and lymphatics to the often hypoxic and acidic tumor microenvironment can have a major impact on T cell metabolism and their resulting effector function (31). Naïve and T_MEM_ cells circulate continuously among the blood vessels, lymph vessels, and lymphatic tissue, whereas activated T cells must suspend migration in the lymphoid tissue to proliferate and then regain their motility to migrate to the tumor (32). Naïve and T_MEM_ cells express CD62L, CCR7, and sphingosine-1-phosphate (S1P), which allows homing to and retention in secondary lymphoid organs (33) (Figure 1). Activation of the PI3K-Akt-mTOR pathway down regulates CD62L, CCR7, and S1P (34). Activation of this pathway also leads to up-regulated expression of pro-inflammatory adhesion molecules such as very late antigen-4 (VLA-4) and chemokine receptors such as CXCR3 (32). These changes allow T_EFF_ cells to migrate to areas of inflammation and might influence their prevalence in visceral adipose tissue (VAT), where they play a pathological role in driving obesity-associated inflammation (9, 33).

### Memory T Cells

Following resolution of an immune response most T_EFF_ cells undergo apoptosis while some remain as T_MEM_ cells, responsible for enhanced immunity against future re-exposure to invading pathogens or tumors (35). While T_EFF_ cells proliferate rapidly and are short-lived, T_MEM_ cells have lower turnover rates. This long life span of T_MEM_ cells poses a unique metabolic demand on these cells. The production of a stable pool of T_MEM_ cells is reliant on a corresponding decrease in mTOR signaling and a shift to fatty acid oxidation (FAO) (36). T_MEM_ cells unlike naïve and T_EFF_ cells have a greater spare respiratory capacity (SRC) in their mitochondria (37) (Figure 1). This allows for rapid ATP production, which gives a bioenergetic advantage upon secondary exposure to its antigen (38). IL-15 increases SRC by enhancing mitochondrial biogenesis and the expression of carnitine palmitoyltransferase I (CPTI). CPTI is a key enzyme involved in FAO. Since FAO takes place in the mitochondria, T_MEM_ cells can increase their use of fatty acids for energy via OxPhos, with a consequential decrease in dependence on glycolysis. This results in T_MEM_ cells having a survival advantage over T_EFF_ cells in the absence of pro-glycolysis signals, when the initial priming antigen has been eliminated and cytokines such as IL-2 dissipate (37).

## How the Tumor Microenvironment Can Affect T Cell Metabolism

### Hypoxia

Tumor growth tends to outgrow the developing blood supply resulting in a hypoxic microenvironment (39). This hypoxic environment can have multiple effects on T cell responses. Roman et al. demonstrated that under hypoxic conditions the secretion of effector CD4^+^ cytokines such as IFN-α was increased resulting in an increased immune response (40). In contrast, other studies have found that hypoxia results in an impaired immune response as a result of reduced IL-2 (41). Hypoxia can affect T cell metabolism through the increased production of lactate by tumor cells within the tumor microenvironment. An increase in lactate in the tumor microenvironment results in decreased T cell proliferation and cytokine production, due to decreased glycolysis (42). HIF-1α is a master transcriptional regulator in the response to low O_2_ levels and is also involved in neoplastic pathways, including angiogenesis, cell survival, and treatment resistance (43). Levels of HIF-1α increase in activated T cells and can also be induced by other stimuli under normoxic conditions such as mTOR signaling (44, 45). HIF-1α is responsible for the glycolytic response that occurs downstream of mTORc1 activation (46). With the exception of Th17 cells, the role of HIF-1α in T cell subset differentiation within the tumor microenvironment has not been clearly defined in the literature and focused studies examining the role of HIF-1α on specific T cell subsets are warranted. Some studies examining T cells in general have shown that it can decrease activated T cell numbers through increased apoptosis (47). Other studies have demonstrated opposing results, showing that HIF-1α prevents apoptosis by up-regulating adrenomedullin (48). A few studies have shown that HIF-1α plays an important role in the differentiation of Th17 cells, which are highly glycolytic and are believed to rely more on glycolysis than other T cell subsets (49). Induction of both Th17 and T_REG_ responses are closely related as they share a common requirement for transforming growth factor (TGF)-β. The expression of HIF-1α plays an important role in regulating the balance of Th17/T_REG_ responses as it inhibits differentiation of T_REG_ cells by targeting Foxp3 for proteasomal degradation (50). It also directly regulates Th17 differentiation by activating transcription of RORγt and collaborates with RORγt to activate Th17 signature genes, such as the IL-17 gene during development (50). The up-regulation of HIF-1α results in high levels of glycolysis in Th17 cells by increasing the expression of Glut1 and enzymes such as pyruvate kinase muscle (PKM) (49). Together with the downregulation of mitochondrial oxygen consumption, this prevents the entry of pyruvate into the TCA cycle (51). It is therefore likely that the constant state of low level inflammation present in many tumors may be due, at least in part, to HIF-1α directed Th17 cell differentiation and decreased T_REG_ cell differentiation (50). A Th17 driven immune response has been linked to infection induced colon cancer in mouse models (52) and *Helicobacter Pylori* infection which can induce gastric cancer (53). Research has recently discovered that not all Th17 cells are pathogenic and drive autoimmune tissue injury (54). Development of pathogenic Th17 cells is dependent on exposure to IL 23, which diminishes the production of the anti-inflammatory cytokine IL-10 (55).

### Nutrient deprivation

Reduction of nutrients present in the microenvironment is associated with an impaired anti-tumor immune response (56). Nutrient deprivation inhibits mTOR activity which is vital for T cell metabolism (57). Glucose is essential for T_EFF_ cell survival and proliferation (5), IFN-γ production (58), and cytolytic activity via production of granzyme and perforin (59). T cell proliferation is inhibited in the absence of glucose even when other metabolic substrates such as fatty acids and glutamine are present (58). T cell activation is also dependent on extracellular glutamine (6). Glutamine is converted to glutamate and subsequently to α-ketoglutarate, which enters into the TCA cycle to generate citrate and pyruvate. This process is known as anaplerosis. It replaces the metabolites that are removed from the TCA cycle for the biosynthesis of fatty acids, nucleotides, and proteins allowing the T_EFF_ cells to maintain the integrity of the TCA cycle function (60). Chang et al. demonstrated that lymphoma cells can impose nutrient deprivation on T cells by depleting glucose and glutamine resources. This can lead to decreased release of cytokines, such as IFN-γ, from T_EFF_ cells (61). Arginine is an example of another amino acid which is vital for many T cell functions such as proliferation (62). Research carried out by Rodriguez et al. demonstrated that myeloid derived suppressor cells in the tumor microenvironment express high levels of arginase-1. The resulting lower levels of arginine led to inhibition of T cell receptor expression and antigen specific T cell responses (63). Sequestration of cysteine by myeloid derived suppressor cells is another way in which amino acid deprivation occurs and subsequently results in the inhibition of T cell activation (64). Tumor cells and non-malignant stromal cells can elicit immunosuppressive effects through the expression of amino acid catabolic enzymes, such as indoleamine 2,3-dioxygenase (IDO) which catalyzes the degradation of tryptophan (65). In fact, IDO expression by tumor cells has been shown to correlate with a poor clinical prognosis in several cancers including ovarian (66) and endometrial cancer (67). Elevated IDO expression causes both the depletion of tryptophan and the production of immunosuppressive tryptophan metabolites (68). Such metabolites can impair T cell function (69) and lead to T cell apoptosis (70), thus resulting in less effective anti-tumor T cell responses. Nutrient limitation can also induce autophagy in T_EFF_ cells, as a survival mechanism to generate an intracellular source of nutrients (71). Reduced levels of amino acid or decreased ATP/AMP ratios result in AMPK activation, which phosphorylates the protein kinase unc-51-like kinase 1/2 (Ulk1/2). Activation of Ulk1/2 then initiates autophagy (72). In addition to autophagy, increased metabolic stress due to nutrient deprivation can ultimately lead to T cell apoptosis (73).

### Chronic T cell activation

Chronic T cell activation occurs due to constant antigen exposure and can induce a state of T cell non-responsiveness termed exhaustion. T cell exhaustion is defined by poor effector function, continued expression of inhibitory receptors, and a gene expression profile distinct from T_EFF_ or T_MEM_ cells (74). The tumor microenvironment establishes an immunosuppressive environment in which T cells respond in a similar manner to exhausted T cells in chronic viral infections (75). This may partially explain why tumors continue to grow despite the presence of tumor specific T cells (76). Baitsch et al. examined T cells from metastases in patients with advanced stage III–IV melanoma and found that these T cells exhibited an exhaustion profile and produced insufficient levels of IFN-γ. The T cell exhaustion at the metastatic tumor sites was induced by continuous antigen exposure coupled with inhibitory signals from tumor cells and non-malignant stromal cells (77). The programed death receptor 1 (PD-1) is an immune-inhibitory receptor, which is expressed on chronically activated T cells. Ahmadzadeh et al. found increased expression of this receptor on tumor infiltrating T cells compared with T cells in peripheral tissue and the blood of patients with metastatic melanoma. The augmented PD-1 expression was associated with impaired effector function in these T cells, decreased proliferation, and reduced ability to differentiate into T_MEM_ cells (76). Cytotoxic T-lymphocyte antigen-4 (CTLA-4) is another negative regulator of T cells, which is up-regulated following T cell activation and during T cell exhaustion (78). Higher levels of CTLA-4 expression on T cells out competes CD28 for binding to CD80 and CD86 on the antigen presenting cell, thus preventing co-stimulation of the T cell (79). CTLA-4 and PD-1 also participate in the regulation of cellular metabolism by blocking CD28-mediated metabolic changes following T cell activation. Ligations of CTLA-4 and PD-1 have been shown to inhibit PI3K/Akt/mTOR signaling resulting in diminished glucose uptake and glycolytic rate (80). Binding of CTLA-4 expressed on T_REG_ cells to CD80 and CD86 on dendritic cells is believed to suppress T cell responses through the up-regulation of IDO expression, thus revealing another mechanism through which CTLA-4 can contribute to tumor antigen tolerance.

## Targeting T Cell Metabolism; A New Avenue for Cancer Immunotherapy

Dysregulation of T cell subsets can result in many immune-mediated disorders, due to a disruption in the balance between protective immunity and suppression of pathological inflammation (1). Modulation of immune cell metabolism has been investigated in conditions where there is a chronic overreaction of the immune system, such as asthma. Chronically activated CD4^+^ T cells isolated from asthma patients have been shown to overexpress pyruvate dehydrogenase kinase-1 (PDK-1), which results in the production of high levels of lactate which promotes aerobic glycolysis. Treatment with the PDK-1 inhibitor dichloroacetate (DCA) inhibited the proliferation of T cells and reduced inflammatory cytokine production by switching the metabolism back to OxPhos. In mouse models this resulted in reduced airway inflammation (81). This study, together with others, has attempted to dampen excessive inflammatory responses through attenuation of T_EFF_ cell responses and enhancement of T_REG_ cell responses by targeting metabolic pathways. Treatment of T cells isolated from a mouse model of asthma with metformin induced the generation of T_REG_ cells through the activation of AMPK and therefore, conferred protective immunity without inappropriate inflammation (28). Metformin is an oral anti-diabetic drug, which reduces hepatic glucose production and increases insulin sensitivity and glucose uptake by muscles and adipocytes, resulting in decreased insulinemia and insulin sensitivity (82). Zhou et al. demonstrated that following inhibition of complex I of the mitochondrial respiratory chain, metformin induces a decrease in ATP synthesis and increases the AMP/ATP ratio, thus stimulating AMPK (83). Studies targeting T cell metabolism have already been conducted in mouse models of arthritis, lupus, uveitis, and asthma and similar studies in obesity-associated cancer might elucidate a mechanism by which the T cell inflammatory responses can be abrogated in adipose tissue to attenuate tumor-promoting chronic inflammation (84–87). Targeting T cell metabolism for cancer immunotherapy must take a different approach by augmenting the beneficial anti-tumor responses of T_EFF_ cells initially, leading to T_MEM_ cell generation and by attenuating the responses of T_REG_ cells. A detailed understanding of the exact metabolic needs of different T cell subsets will allow therapies to be designed in order to achieve this (27).

### Decreasing T_REG_ cell proliferation

**Figure 2:**
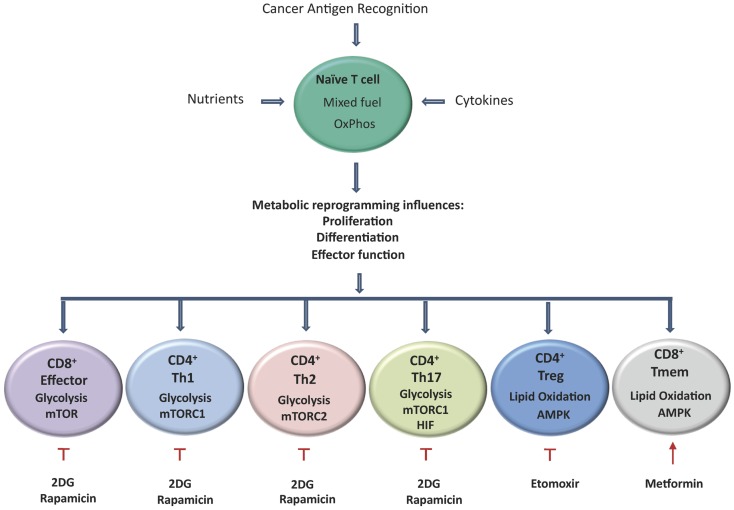
**Activation of T cells results in metabolic reprograming, which influences their proliferation, differentiation, and effector functions**. Aerobic glycolysis becomes the predominant metabolic pathway used by CD8^+^ effector cells, CD4^+^ Th1, Th2, and Th17 cells, by contrast T_REG_ and T_MEM_ rely on lipid oxidation. T cells that differentiate into Th17 cells primarily rely on glycolysis controlled by HIF-1α. T_MEM_ and T_REG_ rely on lipid oxidation under the control of AMPK. Tumor specific T_MEM_ cell prevalence can be enhanced through the inhibition of glycolysis following treatment with the hexokinase-2 inhibitor 2DG. In addition, inhibition of mTOR by rapamycin and activation of AMPK by the mitochondrial complex I inhibitor metformin can also led to increased survival of T_MEM_ cells. Etomoxir, a specific inhibitor of CPT1A results in the reduction of T_REG_ prevalence and has also shown promising results in delaying tumor development.

Increased numbers of T_REG_ cells within the tumor microenvironment have been reported in patients with cancer (88) and are associated with a poorer outcome (89). One theory proposed is that tumor cells both secrete and induce the secretion of TGF-β/IL-10 from immature dendritic cells and results in the generation of T_REG_ cells from CD4^+^CD25^−^ naïve T cells (90). It has been demonstrated in mouse models of cancer that depletion of T_REG_ cells improves endogenous immune-mediated tumor rejection (91). Clinical trials have shown the feasibility and relative safety of managing T_REG_ cells in human cancer, although treatment effects have been modest. Ontak is a monovalent human IL-2 fusion toxin licensed to treat human CD25^+^ cutaneous T cell lymphoma (92). Due to the phenotypic similarity of CD4^+^CD25^+^ cutaneous T cell lymphoma cells and CD4^+^CD25^+^ T_REG_ cells, Ontak was tested in many cancers. Some studies demonstrated depletion in T_REG_ cell numbers and improved immunity in patients with metastatic ovarian carcinoma (93), while other studies showed no benefit in melanoma patients (94). Modulation of T_REG_ cell metabolism to suppress their differentiation is a novel approach being investigated *in vivo*. T_REG_ cells require lipid oxidation for differentiation and treatment of these cells with etomoxir, a CPT1A blocker, has been successful in suppressing T_REG_ cell generation (Figure 2). This study is very promising for cancer therapy due to the fact that T_REG_ cell generation was decreased but the development of IFN-γ secreting Th1 cells was not affected (28).

### Increasing T_EFF_ cell proliferation

Perhaps the most beneficial approach to boost anti-tumor immunity is to increase activated tumor specific T_EFF_ cell numbers (Figure 2). The development of IDO inhibitors is one avenue currently being explored for reactivation of T cells which have become exhausted or tolerant to tumor antigens. One of the most widely studied IDO inhibitors is 1-methyl-tryptophan. In combination with other chemotherapeutic agents such as cyclophosphamide, it led to the regression of established tumors in a mouse model of HER-2 breast cancer (95). Another novel IDO inhibitor: INCB024360 inhibited tumor growth in cell and mouse models due to increased T cell proliferation and IFN-γ production (96). Reactivating T_EFF_ cells by increasing tryptophan levels is advantageous over reactivation by increasing glycolysis, due to the similar glycolytic metabolic pathways used by both T cells and tumor cells for cell survival and growth (97). Doedens et al. have demonstrated that genetically activating the HIF-1α pathway in a B16 mouse melanoma tumor cell line can prevent T_EFF_ cell exhaustion by sustaining the effector functions of T cells despite persistent antigen exposure (98). As previously discussed, the HIF-1α pathway can serve as both a positive and negative regulator of T_EFF_ cells. Further research is required to elucidate the net effect of using HIF-1α activators to boost T_EFF_ cell-mediated responses to persistent tumor antigen challenge. Blockade of CTLA-4 by antibodies prevents the inhibitory signal to the T cell resulting in an enhanced T cell response against tumor cells and improved anti-tumor activity (79). Ipilimumab is a fully humanized IgG1 monoclonal antibody against the extracellular domain of CTLA-4 and has been approved for the treatment of metastatic melanoma (99). Ipilimumab binding to CTLA-4 allows CD28 on the T cell to bind to the co-stimulatory molecules CD80 and CD86 on the antigen presenting cell. This is cited as the mechanism of action of ipilimumab (99). CTLA-4 also inhibits increased glucose metabolism following T cell activation which is highly relevant as increased glucose metabolism is vital to sustain the increased metabolic demands that occur in the transition of a naïve T cell to a T_EFF_ cell (80, 100). Therefore, it can be concluded from this evidence that the success of ipilimumab in the treatment of malignant melanoma (101) might be in part due to its negative effects on glucose metabolism. Some drug therapies currently available affect T cell metabolism as well as their intended therapeutic targets. Imatinib is a tyrosine kinase inhibitor originally designed to target Bcr-Abl in patients with chronic myeloid leukemia (102). However, it has been shown to have opposing effects on T cell metabolism. Balachandran et al. found that imatinib can activate CD8^+^ T cells and induce T_REG_ cell apoptosis in gastrointestinal tumors through downregulation of IDO, thus resulting in augmented anti-tumor activity (103). Others found that imatinib inhibits both normal and leukemic T cell response through the inhibition of Lck-mediated T cell receptor signaling (104, 105). T cell receptor signaling is necessary for maximum glucose uptake following TCR stimulation, through elevated expression and cell surface trafficking of the glucose transporter Glut1. Without correct T cell receptor signaling, T cells cannot correctly adjust their metabolic profile to become T_EFF_ cells and in this manner, imatinib might be detrimental to T cell mediated anti-tumor immune responses (58).

### Increasing T_MEM_ cell prevalence

Increasing the number of long-lived tumor specific T_MEM_ cells can lead to sustained anti-tumor activity due to their increased survival and proliferative capacities. In a study by Sukumar et al., the glucose analog 2-deoxyglucose (2DG), an inhibitor of hexokinase-2 (Hk2), was used to block glycolysis (Figure 2). This resulted in the increased activity of signaling pathways and transcription factors that enhance T_MEM_ cell development, such as AMPK which negatively regulated mTOR and Foxo1, and resulted in enhanced anti-tumor activity of CD8^+^ T cells (106). Other methods to increase the generation of T_MEM_ cells by the modulation of fatty acid metabolism have also been investigated. Treatment with metformin to activate AMPK promoted T_MEM_ cell generation and was able to significantly improve the efficacy of an experimental anti-cancer vaccine *in vivo* (10) (Figure 2). Metformin has already been shown to inhibit tumor formation due to its direct action on the tumor, but its use as a potentiator of T_MEM_ cell activity still requires further investigation (107). Many mechanisms of action have been proposed for the anti-cancer properties of metformin, including its actions on mTOR (108) and novel actions such as up-regulation of miR33a resulting in down regulated c-Myc expression (109). Rapamycin, through the inhibition of mTOR (specifically complex 1) reduces glycolysis and increases lipid oxidation and can also enhance the formation of T_MEM_ cells (36) (Figure 2). Other modes of action investigated for rapamycin-induced development of T_MEM_ cells include the inhibition of the Th1 promoting transcription factor T-bet and the stabilization of the expression of the related transcription factor eomesodermin (110). Treatment of mice with rapamycin following viral infection not only enhanced frequencies but also accelerated the transition from T_EFF_ cells to virus specific T_MEM_ cells without inhibiting the ability of the infected animal to clear the antigen (111). Due to the multiple actions of mTOR, rapamycin can potentially exert multiple effects on T cell metabolism, particularly its rapid induction of autophagy, which can be both a pro- and anti-survival process in T cells (112). Like metformin, rapamycin can directly target and inhibit tumor cell growth in many ways such as induction of apoptosis (113) and inhibiting angiogenesis (114). Unlike metformin however, rapamycin has immunosuppressive properties and is used as anti-rejection therapy due to its anti-proliferative actions (115). Although to date, the anti-cancer effects of rapamycin and its derivatives are more prominent than the immunosuppressant effects, further research is warranted to ensure this is also the case in the long term (116). Another important role for any drugs developed to enhance the formation of T_MEM_ cells is to enhance the efficacy of cancer vaccines. The main goal of any vaccination is to induce an effective T_MEM_ cell response (117), many of the experiments targeting T_MEM_ cell generation have a better recall response and are more protective following re-challenge (111).

### Targeting T cell metabolism to prevent and treat obesity-associated cancer

Obesity induces a state of tumor-promoting chronic inflammation, marked by elevated levels of free fatty acids, abnormal cytokine production, and activation of inflammatory signaling pathways (118, 119). The expansion of VAT in obesity, results in increased blood supply, inflammatory immune cell infiltration, and enhanced pro-inflammatory cytokine release (9, 120–122, 126). Our group have previously shown that activated IFN-γ producing CD4^+^ and CD8^+^ T cells are significantly enriched in obese VAT, while others have found that frequencies of T_REG_ cells are decreased (9, 123, 124). Therefore, obese VAT is characterized by an increased prevalence of pro-inflammatory T cells and reduced numbers of anti-inflammatory T cells, which is likely to contribute to adipose tissue inflammation and the clinical manifestations of obesity, including cancer. Developing effective immunotherapies for obesity-associated malignancies represents a unique challenge; to attenuate chronic systemic inflammation while simultaneously augmenting specific immunity at tumor sites. As pro-inflammatory Th1 cells appear to play a crucial role in obesity-associated inflammation, the alteration of the metabolic profile of T cells in the VAT may present a favorable therapeutic option. There is currently a paucity of studies investigating the efficacy of targeting immunometabolism as a mechanism to control tumor-promoting systemic inflammation associated with obesity. However, there is some precedence for targeting metabolism to control systemic inflammation in autoimmune diseases. For instance, inhibition of both the mTOR and MAPK (ERK) pathways using rapamycin and the MEK1/2 inhibitor PD325901 was identified as a means of robustly blocking effector CD4^+^ T cell proliferation in a mouse model of arthritis (84). This study found that inhibition of both pathways was more effective than inhibiting either mTOR or ERK alone and resulted in greatly decreased disease severity in a mouse model of arthritis. Since our group has previously identified T_EFF_ cells as key players in adipose tissue inflammation, ERK and mTOR inhibitors might elicit a similar anti-inflammatory effect in obesity (9). Others have found that beauvericin-mediated inhibition of T cell activation in colitis was achieved by targeting P13K and AKT (86). Treatment with beauvericin reduced pro-inflammatory cytokine production, weight loss, diarrhea, and mortality in this mouse model of colitis (86). In addition, pro-inflammatory cytokine production and clinical symptoms in experimental autoimmune uveitis (EAU) were reduced through the inhibition of PI3K/AKT by a novel anti-angiogenic peptide H-RN, thus presenting another potential therapeutic option for attenuating obesity-associated inflammation (87). The small molecule inhibitor IPI-145 has also shown promising therapeutic value in the blockade of PI3K in collagen-induced arthritis, ovalbumin-induced asthma, and systemic lupus suggesting that this molecule might be applicable and effective in autoimmune and multiple inflammatory disorders, including obesity (85). The inhibitor elicited its effects through blockade of both PI3K-δ and PI3K-γ and subsequent suppression of both innate and adaptive immune responses. This resulted in reduced pro-inflammatory cytokine production and disease severity in all mouse models tested (85). From these studies, it appears that anti-inflammatory agents targeting PI3K, AKT, and mTOR could be a favorable option in the treatment of obesity-associated inflammation and cancer. However, metabolic targets for the treatment of obesity-associated cancer and disease should first be identified through definitive studies that will elucidate the metabolic profiles of T cells in VAT and the corresponding tumor. A therapy targeting specific metabolic pathways of VAT T cells could be used alone as a prophylactic in obese individuals who are at risk of developing malignancy or in combination with standard therapies in patients who have already developed an obesity-associated malignancy. Focused studies are currently lacking but clearly warranted in this area.

## Conclusion

It is clear that T cell survival, function, and differentiation are intimately linked to T cell metabolism. The increase in glycolysis and glutaminolysis facilitate the rapid proliferation of T cells required during activation to allow fast clearance of pathogens or tumor cells. T_REG_ and T_MEM_ cells favor FAO, which allows these cells to survive long term, when glucose and growth factors are limited. This ensures the control of inappropriate immune responses and long term immunity. Extensive metabolic reprograming not only fulfils the extra biosynthetic demands of activated T cells, but it is also integrated with signaling pathways that determine T cell fate, thus enhancing our understanding of distinct T cell subset function and regulation. The different metabolic pathways may also provide a new opportunity for modulating the immune response during tumor development and progression. One of the hallmarks of cancer is evasion of the immune system (125) and in the past few years our understanding of how this occurs has greatly improved. One area which holds promise for cancer immunotherapy is the manipulation of immune responses, such that T_EFF_ and T_MEM_ cell-mediated responses are enhanced, while those of T_REG_ cells are decreased, ultimately providing a therapy that might “switch” back on the immune system to target the tumor cell. Reactivating exhausted T cells by adjusting their metabolism also holds potential as a treatment for cancer, however more research needs to be carried out to fully elucidate the role of cellular metabolism in T cell exhaustion. Due to the similarities between tumor cells and activated T cell’s metabolic programing, any therapies designed to modulate tumor cell metabolism must take into account their effects on T cell metabolism in the immune response to cancer.

Since the WHO now estimates that obesity is directly attributable for as many as 41% of malignancies and with the rates of obesity continuing to increase dramatically, it is clear that novel therapeutic options are urgently required. Since activated inflammatory T_EFF_ cells are found to be enriched in the VAT of cancer patients, it is likely that they play pathological roles in driving obesity-associated inflammation. Attenuation of this inflammation might be achieved via metabolic manipulation of inflammatory T_EFF_ cell differentiation and activation. Suppression of excessive pro-inflammatory T cell responses through inhibition of metabolic pathways has already been demonstrated across a spectrum of chronic inflammatory disorders in mice and could represent a viable therapeutic target for obesity-associated cancers.

The possibilities of modulating anti-tumor immune responses through targeting T cell metabolic pathways hold huge promise and clearly warrant further investigation.

## Conflict of Interest Statement

The authors declare that the research was conducted in the absence of any commercial or financial relationships that could be construed as a potential conflict of interest.
